# On the Use of Popular Basis Sets: Impact of the Intramolecular Basis Set Superposition Error

**DOI:** 10.3390/molecules24203810

**Published:** 2019-10-22

**Authors:** Ángel Vidal Vidal, Luis Carlos de Vicente Poutás, Olalla Nieto Faza, Carlos Silva López

**Affiliations:** 1 Departamento de Química Orgánica, Facultade de Química, Universidade de Vigo, 36310 Vigo, Spain; a.vidal.vidal@uvigo.es (Á.V.V.); lcpoutas@gmail.com (L.C.d.V.P.); 2 CITACA, Agri-Food Research and Transfer Cluster, Campus da Auga, University of Vigo, 32004 Ourense, Spain; faza@uvigo.es; 3 Departamento de Química Orgánica, Facultade de Ciencias, Universidade de Vigo, 32004 Ourense, Spain

**Keywords:** BSSE, proton affinity, basis set errors, relative energies

## Abstract

The magnitude of intramolecular basis set superposition error (BSSE) is revealed via computing systematic trends in molecular properties. This type of error is largely neglected in the study of the chemical properties of small molecules and it has historically been analyzed just in the study of large molecules and processes dominated by non-covalent interactions (typically dimerization or molecular complexation and recognition events). In this work we try to provide proof of the broader prevalence of this error, which permeates all types of electronic structure calculations, particularly when employing insufficiently large basis sets.

## 1. Introduction

**Figure 1:**
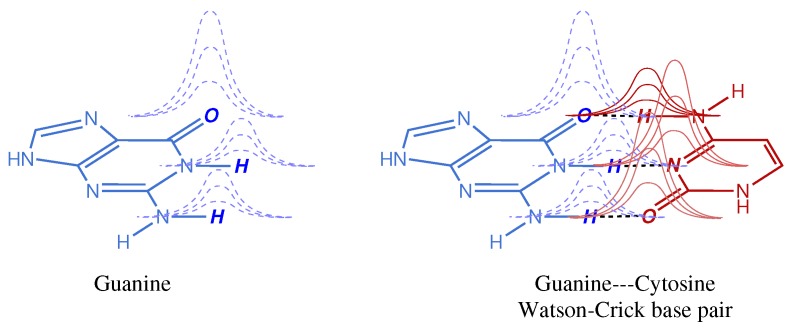
Schematic representation of the basis set superposition error (BSSE) in a single Watson–Crick base pair. Atom centered Gaussian functions are represented with like colors, atoms whose electron density is partially described by Gaussian tails of functions centered in atoms belonging to the other monomer are indicated in boldface and slanted typography.

Basis set superposition error (BSSE) is a fundamental issue in electronic structure calculations [1]. Its academic definition is usually based on the monomer/dimer dichotomy: *In an electronic structure calculation, the energy contribution of each monomer to the dimer is artificially shifted down with respect to that of the isolated monomer due to the stabilizing effect of overlapping basis belonging to the other monomer*. This problem is indissoluble with the use of atom centered basis sets (which are usually Gaussian functions) but it is worth noting that alternatives, such as the use of plane waves as basis sets, exist in which the BSSE is avoided. A simple illustration of the intermolecular BSSE in a biologically relevant dimer can be shown in Figure 1. The effect of Gaussian tails overlapping with atoms of a different monomer is intrinsically minor in absolute magnitude, but it may have quite an impact when these small contributions accumulate in a molecular or macromolecular system. For instance, the BSSE of a single Watson–Crick base pair may be negligible for most chemical purposes (depending on the basis set used) but it adds up quickly as the system size increases. Moreover, in weak interactions, like those found not only in DNA base pairs but also in host–guest complexes, both natural and artificial, this error cannot be ignored because it may account for a large fraction of the weak interaction being computed.

Complexes held together by non-covalent interactions were probably the first and foremost systems where this error was accounted for. Two reasons could be brought to the table for this: (1) The ability to compute the energy of the separate vs. joined monomers and (2) the relatively easy methodology to avoid this error. Both reasons are actually interconnected and their connection can be easily understood through the proposed method to solve the BSSE in non-covalently bonded complexes: the counterpoise correction by Boys and Bernardi [2]. This scenario has facilitated the spreading of the idea that the BSSE is an issue to take into account only when dealing with molecular recognition processes, host–guest complexes, or dimerization reactions. As a result, vast literature is available in which the BSSE is studied or dealt with in intermolecular complexes, to such an extent that not only the existence of this error is discussed with amplitude, but also the appropriateness of the most commonly employed correction method indicated above [3,4,5,6,7]. Despite the general idea that this error is contingent to weak interactions, seminal work on basis set development warned about this problem also affecting strong interactions (covalent bonds). This early concern for BSSE through covalent bonds is closely related to the development of Atomic Natural Orbital (ANO) basis sets and the study of small molecules with strongly correlated methods [8]. The reason behind for this lies in the fact that when the covalent bond is cleaved in diatomic molecules, the final fragments are atomic entities and ANO basis sets are particularly well suited for the description of their electronic structure [8,9,10]. This idea however has remain mostly restricted to those involved in basis set development and highly accurate simulations of small molecules (through coupled cluster or MRCI methods). The intramolecular BSSE is however common to any methodology, whether derived from Hartree–Fock or from density functional theory. The popularization of the latter as an efficient tool for a plethora of computational chemistry applications happened, leaving this problem behind until relatively recently. Some authors have therefore called on the idea that the BSSE should be taken into consideration also from an intramolecular perspective (and hence in covalent interactions) in essentially all kinds of electronic structure calculations when relative energies are computed, which they are in the vast majority of occasions. The definition of the BSSE should, therefore, be written in more general terms. An example of this has been recently proposed by Hobza: *“The BSSE originates from a non-adequate description of a subsystem that then* tries *to improve it by* borrowing *functions from the other sub-system(s)”* [11]. He continues the description by clarifying how the BSSE can be understood in intramolecular terms: *“the same effect should take place also within an isolated system where one part is improving its description by* borrowing*orbitals from the other one”.*

Interestingly Hobza also mentions how the existence of this error has been largely denied in its intramolecular version and only recently *discovered*. This late discovery was triggered by researchers reporting shocking results based on electronic structure calculations with basis sets of limited size. An example of this kind of results is the non-planar benzene (among other heterocycles) reported by Schaefer et al. [12]. Salvador et al. provided evidence that these anomalous results in the geometries of a number of arenes stemmed from intramolecular BSSE [13]. Before these mind-changing reports were published, a number of authors had already revealed clear cases of intramolecular BSSE, and they had actually shown that this error is not consigned to larger systems, as small molecules such as F${}_{2}$, water, or ammonia were affected [14,15,16,17]. Combining the original idea of the BSSE belonging to processes involving separate fragments and considering that these fragments could be interacting by incipient covalent bonds Dannenberg et al. showed how results on the transition state of the paradigmatic Diels–Alder reaction could also suffer from this error [18,19].

Most of the studies mentioned above use molecular geometries to highlight the presence of intramolecular BSSE in their calculations. In some cases, conformational energies are also used as diagnostic parameters to highlight the effects of this error. These findings, however, have not permeated to the broad community employing electronic structure calculations. In an attempt to convey the importance of this effect to the wider community we decided to showcase this effect in a systematic series not involving structure, but chemical reactivity. An appropriate kind of reactivity to illustrate this problem should verify a number of conditions:

- It should be experimentally operative in the gas phase, in order to avoid artifacts stemming from the effect of the environment.
- Accurate experimental data should be readily available.
- This chemistry should span a relatively wide range of energies, in order to maximize the signal/noise ratio.
- Structural changes should ideally be systematic in the sense of involving an increasing number of basis set functions without adding strong electronic or steric effects that could obscure the pure dependecy on these functions.
- Ideally, the molecular change upon reaction should be very local to have a clear *locus* on which the BSSE acts.
- If possible, precedents on the succesful description of the reactivity of choice by computational methods should be available.

After revising all these conditions, and taking into account our previous experience in benchmarking density functional methods in terms of their accurary with respect to the calculation of proton affinities, we decided to use a systematic series of hydrocarbons of increasing size and analyze how the BSSE affects their proton affinities and gas-phase basicities when employing basis sets of different sizes [20]. This reactivity verifies all the above conditions, and it is fundamental in such a way to chemistry that substantial effort has been devoted exclusively to computationally describe it in high accuracy [21,22,23,24,25]. It is worth noting however, that the target of the present work will not be such high accuracy in the calculation of proton affinities, but exposing the effects of intramolecular basis set superposition error (BSSE) and basis set incompleteness error (BSIE). These two errors will be revealed in orthogonal directions as the size of the basis set and the size of the molecular system are varied, in a way that could be likely described as *two faces of the same coin*.

## 2. Theoretical Background and Computational Methods

A simple example of reactivity (proton affinity and gas-phase basicity) has been chosen for which very accurate experimental values are available in the literature, to analyze the effect of BSSE and BSIE. Both magnitudes are related to the same gas-phase reaction:

(1) $$B^{-}+H^{+}\to B-H.$$

While the proton affinity (PA) is defined (IUPAC) as *“The negative of the enthalpy change in the gas phase reaction between a proton and the chemical species concerned, usually an electrically neutral species, to give the conjugate acid of that species”*, the gas-phase basicity (GPB) is defined as the negative of the Gibbs free energy change associated with the previously described reaction.

Density functional theory in the Kohn–Sham formulation as implemented in Gaussian 16 [26] was used to locate minimum structures on the potential energy surfaces of the systems under study. To achieve results with high accuracy, a superfine pruned grid for the numerical integration containing 150 radial points and 974 angular points per shell was used in combination with a tight self-consistent field (SCF) convergence criteria. Harmonic analysis of the second derivatives of the energy with respect to the nuclear displacements were also computed for each stationary point to ensure that a minimum energy structure and not a transition state or higher order saddle point was located. The required thermodynamic properties for the computation of PA and GPB were obtained from the electronic structure, density functional theory calculations, using standard statistical mechanical expressions for separable vibrational, rotational, and translational contributions within the harmonic oscillator, rigid rotor, and ideal gas/particle-in-a-box models in the canonical ensemble [27]. The standard state in the gas phase was for a mole of particles at 298.15 K and 1 atm pressure. No scaling factor for the frequencies was used for the calculations.

The entropy of the proton is obtained using the Sackur–Tetrode equation derived from statistical thermodynamics [28,29]:

(2) $$S\left(H^{+}\right)=Rln\left(\frac{e^{\frac{5}{2}}k_{B}T}{p\Lambda^{3}}\right),$$

where Λ is known as the thermal De Broglie wavelength and is defined as:

(3) $$\Lambda ={\left(\frac{h^{2}}{2\pi mk_{B}T}\right)}^{1/2}.$$

Note that on the previous equations, $k_{B}$ is the Boltzmann constant, p the pressure, *T* the absolute temperature, *h* the Planck constant, and *m* the mass of the proton. Moreover, the gas-phase enthalpy of the proton was derived from the ideal gas law:

(4) $$H\left(H^{+}\right)=U+PV=\frac{5}{2}RT,$$

where *P* and *V* are the pressure and volume respectively, *U* the internal energy, *T* the absolute temperature, and *R* the universal gas constant.

Using the equations shown above, the values for the entropy and enthalpy in the standard state are: 26.02 cal/mol·K and 1.48 kcal/mol under standard state conditions. The combination of both quatities gives the gas phase Gibbs free energy for the proton as:

(5) $$G\left(H^{+}\right)=H\left(H^{+}\right)-TS\left(H^{+}\right).$$

Two different density functionals were used for the calculation of the PA and GPB: ωB97XD and mPW1B95 [30,31]. The first is a multiparameter meta-hybrid from Head Gordon and coworkers. This functional includes 100% long-range exact exchange, around a 22% of short-range exact exchange and also a modified B97 exchange density functional for short-range interactions. It also uses the B97 correlation formula and empirical dispersion corrections. On the other hand, mPW1B95 is a one parameter hybrid functional that uses the Barone’s modified Perdew–Wang (mPW) exchange and B95 correlation functional. The mPW1B95 functional has been chosen due to the good performance in the calculation of proton affinities compared with high accurate multilevel methods such as CBS-QB3, G3B3, and G3MP2B3 reported in previous publications [32,33,34,35]. Likewise, it has been considered very interesting to include in the study a multiparametrized density functional to compare the performance of both with respect to the BSIE and the BSSE. Six different growing size (in terms of primitive functions) Slater and Pople basis sets were used with both functionals to obtain PA and GPB values: STO-3G **(b0)** [36,37], 3-21G **(b1)** [38,39,40], 6-31G(d) **(b2)** [41,42,43,44,45], 6-31+G(d,p) **(b3)** [46,47,48], 6-311+G(d,p) **(b4)** [49,50] 6-311++G(3df,2pd), **(b5)** [51,52,53,54]. The reasoning behind this choice of basis sets is both their popularity and the fact that they span a very wide range in terms of the number of functions per atom, allowing us to better highlight the BSSE and BSIE effects.

## 3. Results and Discussion

**Figure 2:**
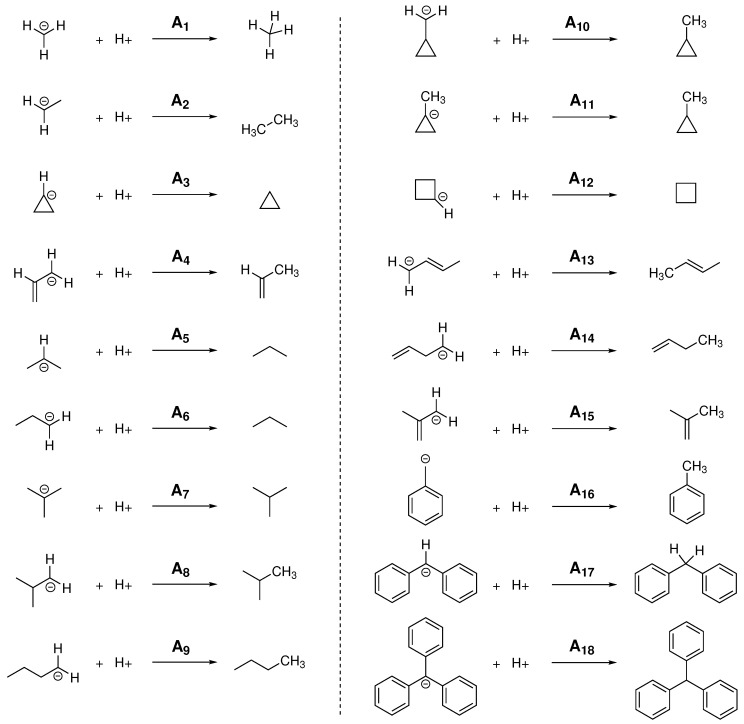
Schematic representation of the 18 different reactions used in this study for the calculation of proton affinity (PA) and gas-phase basicity (GPB).

For the calculation of PA and GPB, different linear and branched alkanes have been chosen considering, in some cases, different possible protonation sites within the molecule, obtaining in this way the 18 reactions shown in Figure 2. Experimental values for PAs and GPBs have been taken from the NIST Standard Reference Database [55,56].

To facilitate the visualization of the results, the relative errors (expressed as a percentage with respect to the experimental reference value) have been calculated. The results are shown in Table 1 for the PAs and in Table 2 for the GPB, using in both cases a red–yellow–green color scale as the computed values improve (lower errors in green) with respect to the experimental reference. These tables should be analyzed from left to right and also from top to bottom, since they contain information about both, the BSSE (top to bottom) and the BSIE (left to right). Absolute values of the computed PAs and GPBs can be found in the supporting information.

All systems are ordered according to the number of primitive functions associated to the basis set chosen in each calculation. If the data is analyzed from top to bottom, the BSSE is revealed since there are a greater number of primitive functions in the calculation due to a larger size of the molecular system being computed. In this case, as the molecule becomes larger the chances of atoms involved in the reaction *borrowing* Gaussian functions from contiguous atoms, and thus causing an artificial overstabilization, increases. Since the reaction studied involves structural changes at a single atom, we could argue that the observed trend is mainly due to a single atom actually *borrowing* functions from its neighbors in order to describe better the protonation process. In other words, the atom being protonated has a better effective basis set as the system size increases. In this direction the effects are more pronounced the smaller the basis set. All the results are quite systematic, in the sense that the same trends are observed of both PA and GPB, and for both density functionals. For the smallest basis set, STO-3G, this translates into a relative error of about 32% for the protonation of methyl anion whereas the relative error for the trityl anion is significantly reduced in more to 22% or 17%, depending on the density functional. The same clear effect is observed for the next two basis sets, 3-21G and the popular 6-31G(d), although the errors are reduced to single digit figures (from 9%/7% to 3%/1% kcal/mol for ωB97XD/mPW1B95). When reaching larger basis sets that add diffuse and polarization functions, such as in the case of 6-311++G(3df, 2pd) (**b5**) this systematic error is greatly dilluted and the errors obtained in the calculations have a larger random component.

On the other hand, if data is analyzed from left to right the size of the system is kept constant whereas the size of the basis set increases, hence revealing the effects of the BSIE. It should not be forgotten that the BSIE is no more than the difference between the value obtained with a particular basis set and the complete basis set (CBS) value. As the size of the basis set increases, the BSIE should decrease. This error is even more evident than the intramolecular BSSE and, when the size of the basis set is increased, the error abruptly decreases for the first four basis sets studied (b0 to b3). Once the basis set chosen includes several polarization functions and diffuse functions, the error decreases less rapidly, as expected (the BSIE should become asymptotic as the complete basis set limit is approached). As an example, in the first and smaller system, the percentage error for the PA decreases from 31% in STO-3G to 9% and 7% for 3-21G and 6-31G(d), then the errors are slowly reduced reaching values lower than 1% for larger basis sets.

Those effects reveal themselves with great clarity for the two density functionals employed, although it is particularly relevant to mention that the average error values are greater in the case of the multiparameter density functional ωB97Xd than in the single-parameter mPW1B95. This trend is observed in both directions. The mPW1B95 functional with large basis sets tends to underestimate the value of the calculated PA or GPB in contrast to what happens with the smaller basis sets and also in contrast to the behavior of the ωB97XD density functional, in which the values are generally overestimated. The overall performance of the least parameterized functional is better for both the BP and the GPB.

The GPB exhibits a similar behavior to that shown by the PA, and the effects of the BSSE and the BSIE can be also be appreciated there. Analysis of the trends in the GPB values lead to conclusions parallel to those extracted from the PAs. It should be mentioned, however, that the average percentage errors are greater in the calculation of this second property. This is likely not assignable to the density functionals themselves, but to the approximations that are taken for the calculation of the thermal corrections to the electronic energy as mentioned in the methods section.

## 4. Conclusions

These results have clearly revealed the existence of intramolecular BSSE in chemical reactivity, finding that its impact is not negligible when reaction enthalpies and free energies are computed with very popular basis sets (like 6-31G(d) and 6-31+G(d,p)). The goal of this work is to showcase the relevance of this error in chemical reactions and not to focus on how to solve this problem, however, a possible way to deal with this intramolecular BSSE could be based on dividing a single molecule into different fragments and calculating the energy as is done for the evaluation of the intermolecular BSSE using ghost functions in other parts of the molecule. The combination of the different energies computed would provide a BSSE corrected value, however, this strategy presents a number of problems:

- There is no univocal way of dividing the molecule into fragments, and different partitionings would provide different corrections for the BSSE.
- The complex/monomer approach by Bernardi and Boys is based on rigid (single point) calculations whereas chemical reactions involve changes in molecular structure.
- The number of calculations needed to compute the BSSE correction depends on the number of fragments, which could easily scale up quickly to untractable numbers if fine-grain fragmentation is needed to account for the intramolecular BSSE.

These, among other difficulties, are calling for the development of methodology based in Gaussian functions that intrinsically avoids this error by design, not by an aftermarket correction. Some recent efforts on this line have been directed to at least alleviate the BSSE in a systematic maner. For instance, the geometrical counterpoise of Grimme is an atom pair-wise correction that depends only on the molecular geometry and needs no input from the wavefunction. The method has been parametrized to fit to standard Boys and Bernadi counterpoise corrections for Hobza’s S66x8 set of non-covalently bound complexes [57]. DFT-C is an adaptation of the latter by Head–Gordon which can provide an estimate of both inter- and intra-molecular BSSE (currently however it is only available for the def2-SVPD basis set) [58]. Earlier efforts are also noteworthy, particularly those aimed at dealing with the BSSE through an a priori exclusion rather than the more common a posteriori correction, such as the chemical Hamiltonian approach [59,60].

## Figures and Tables

**Table 1:**
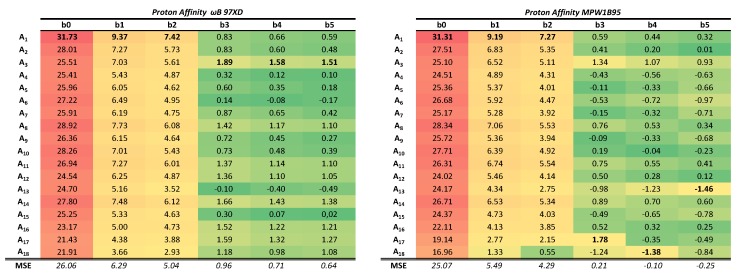
Signed relative error values expressed as percentage with respect to the experimental reference value in the calculation of proton affinities (PAs). The results obtained with different basis sets have been colored following a conditional format and a RYG (red–yellow–green) scale in which the worst results are shown in red while the best are highlighted in green. Each density functional has been colored independently to favor the visualization and comparison of the results. The last row represents the mean signed errors (MSE) for each of the basis sets: **(b0)**: sto-3g, **(b1)**: 3-21g, **(b2)**: 6-31g(d), **(b3)**: 6-31+g(d,p), **(b4)**: 6-311+g(d,p), **(b5)**: 6-311++g(3df,2pd).

**Table 2:**
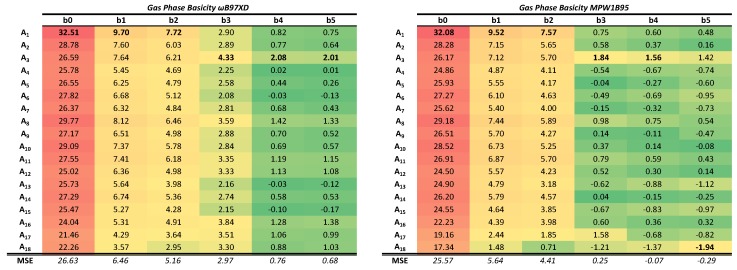
Signed relative errors at each theoretical level and mean signed errors (MSE) for each basis set in the values obtained for the gas-phase basicities (GPBs). The color scale used, method of cell coloring, and basis set employed are the same described for PAs.

## Supplementary Materials

The following are available online. Table S1: Computed values of the proton affinity (kJ/mol) and errors (in percentage) with respect to the experimental result obtained with the MPW1B95 density functional and different basis sets, Table S2: Computed values of the proton affinity (kJ/mol) and errors (in percentage) with respect to the experimental result obtained with the wB97XD density functional and different basis sets, Table S3: Computed values of the gas phase basicity (kJ/mol) and errors (in percentage) with respect to the experimental result obtained with the MPW1B95 density functional and different basis sets, Table S4: Computed values of the gas phase basicity (kJ/mol) and errors (in percentage) with respect to the experimental result obtained with the wB97XD density functional and different basis sets.

## Abbreviations

The following abbreviations are used in this manuscript:

BSSE	Basis set superposition error
BSIE	Basis set incompleteness error
DNA	Deoxyribonucleic acid
ANO	Atomic Natural Orbital
MRCI	Multi-reference configuration interactions
