# Supplementary material for: On the Use of Popular Basis Sets: Impact of the Intramolecular Basis Set Superposition Error
Source: Molecules. 2019 Oct 22;24(20):3810. doi: 10.3390/molecules24203810 (PMC6832644; doi:10.3390/molecules24203810)
Supplement: Supplementary file 1 [file molecules-24-03810-s001.pdf]

On the Use of Popular Basis Sets: Impact of the Intramolecular Basis Set Superposition Error

Ángel Vidal Vidal [1,3], Luis Carlos de Vicente Poutás [1], Olalla Nieto Faza [2,3] and Carlos Silva López [1,3]*

[1] Departamento de Química Orgánica. Facultade de Química. Universidade de Vigo. 36310, Vigo, Spain
[2] Departamento de Química Orgánica. Facultade de Ciencias. Universidade de Vigo. 32004, Ourense, Spain
[3] CITACA, Agri-Food Research and Transfer Cluster, Campus da Auga, University of Vigo, 32004-Ourense, Spain

* Correspondence: carlos.silva@uvigo.es

Contents:

1. **Table 1**: Computed values of the proton affinity (kJ/mol) and errors (in percentage) with respect to the experimental result obtained with the MPW1B95 density functional and different basis sets……1
2. **Table 2:** Computed values of the proton affinity (kJ/mol) and errors (in percentage) with respect to the experimental result obtained with the ωB97X-D density functional and different basis sets ……2
3. **Table 3:** Computed values of the gas phase basicity (kJ/mol) and errors (in percentage) with respect to the experimental result obtained with the MPW1B95 density functional and different basis sets ……3
4. **Table 4:** Computed values of the gas phase basicity (kJ/mol) and errors (in percentage) with respect to the experimental result obtained with the ωB97X-D density functional and different basis sets ……4

Table 1: Computed values of the proton affinity (kJ/mol) and errors (in percentage) with respect to the experimental result obtained with the MPW1B95 density functional and different basis sets.

MPW1B95													
System	Ref. Value	B0		B1		B2		B3		B4		B5	
		Computed	Error (%)	Computed	Error (%)	Computed	Error (%)	Computed	Error (%)	Computed	Error (%)	Computed	Error (%)
**A_1**	1743.6	2289.44	31.31	1903.81	9.19	1870.40	7.27	1753.86	0.59	1751.22	0.44	1749.22	0.32
**A_2**	1758	2241.68	27.51	1878.00	6.83	1852.07	5.35	1765.21	0.41	1761.56	0.20	1758.11	0.01
**A_3**	1716	2146.64	25.10	1827.93	6.52	1803.65	5.11	1739.05	1.34	1734.44	1.07	1732.00	0.93
**A_4**	1636.4	2037.56	24.51	1716.42	4.89	1706.87	4.31	1629.31	-0.43	1627.28	-0.56	1626.04	-0.63
**A_5**	1755	2223.29	26.68	1858.91	5.92	1833.37	4.47	1745.78	-0.53	1742.29	-0.72	1738.00	-0.97
**A_6**	1755	2199.99	25.36	1849.18	5.37	1825.43	4.01	1752.99	-0.11	1749.13	-0.33	1743.36	-0.66
**A_7**	1728	2163.00	25.17	1728.00	5.28	1795.75	3.92	1725.48	-0.15	1722.52	-0.32	1715.73	-0.71
**A_8**	1728	2217.79	28.34	1850.02	7.06	1823.53	5.53	1741.11	0.76	1737.11	0.53	1733.86	0.34
**A_9**	1745	2193.83	25.72	1838.46	5.36	1813.79	3.94	1743.38	-0.09	1739.16	-0.33	1733.09	-0.68
**A_{10}**	1745	2228.50	27.71	1856.45	6.39	1830.93	4.92	1748.27	0.19	1744.32	-0.04	1741.05	-0.23
**A_{11}**	1720	2172.54	26.31	1835.89	6.74	1815.32	5.54	1732.94	0.75	1729.49	0.55	1727.03	0.41
**A_{12}**	1712	2123.29	24.02	1805.52	5.46	1782.85	4.14	1720.56	0.50	1716.80	0.28	1714.13	0.12
**A_{13}**	1757	2181.65	24.17	1833.19	4.34	1805.24	2.75	1739.83	-0.98	1735.32	-1.23	1731.34	-1.46
**A_{14}**	1707	2162.97	26.71	1818.43	6.53	1798.09	5.34	1722.14	0.89	1718.89	0.70	1717.23	0.60
**A_{15}**	1644	2044.69	24.37	1721.72	4.73	1710.17	4.03	1635.95	-0.49	1633.26	-0.65	1631.22	-0.78
**A_{16}**	1593	1945.28	22.11	1658.74	4.13	1654.26	3.85	1601.30	0.52	1598.03	0.32	1596.93	0.25
**A_{17}**	1521	1812.08	19.14	1563.17	2.77	1553.63	2.15	1548.06	1.78	1515.75	-0.35	1513.60	-0.49
**A_{18}**	1501	1755.61	16.96	1521.00	1.33	1509.22	0.55	1482.33	-1.24	1480.36	-1.38	1488.39	-0.84

Table 2: Computed values of the proton affinity (kJ/mol) and errors (in percentage) with respect to the experimental result obtained with the ωB97X-D density functional and different basis sets.

ωB97X-D													
System	Ref. Value	B0		B1		B2		B3		B4		B5	
		Computed	Error (%)	Computed	Error (%)	Computed	Error (%)	Computed	Error (%)	Computed	Error (%)	Computed	Error (%)
A_1	1743.6	2296.77	31.73	1906.96	9.37	1873.00	7.42	1758.12	0.83	1755.11	0.66	1753.87	0.59
A_2	1758	2250.45	28.01	1885.79	7.27	1858.66	5.73	1772.58	0.83	1768.49	0.60	1766.35	0.48
A_3	1716	2153.81	25.51	1836.67	7.03	1812.27	5.61	1748.42	1.89	1743.08	1.58	1741.93	1.51
A_4	1636.4	2052.20	25.41	1725.22	5.43	1716.16	4.87	1641.56	0.32	1638.32	0.12	1638.08	0.10
A_5	1755	2232.79	27.22	1868.92	6.49	1841.82	4.95	1757.46	0.14	1753.67	-0.08	1752.01	-0.17
A_6	1755	2210.68	25.96	1861.22	6.05	1836.08	4.62	1765.53	0.60	1761.18	0.35	1758.11	0.18
A_7	1728	2175.74	25.91	1835.02	6.19	1810.05	4.75	1743.05	0.87	1739.30	0.65	1735.25	0.42
A_8	1728	2227.75	28.92	1861.56	7.73	1833.15	6.08	1752.55	1.42	1748.28	1.17	1746.99	1.10
A_9	1745	2205.00	26.36	1852.27	6.15	1825.90	4.64	1757.51	0.72	1752.83	0.45	1749.75	0.27
A_{10}	1745	2238.12	28.26	1867.33	7.01	1839.78	5.43	1757.79	0.73	1753.43	0.48	1751.73	0.39
A_{11}	1720	2183.41	26.94	1845.03	7.27	1823.35	6.01	1743.55	1.37	1739.56	1.14	1738.94	1.10
A_{12}	1712	2132.08	24.54	1818.95	6.25	1795.40	4.87	1735.31	1.36	1730.78	1.10	1729.96	1.05
A_{13}	1757	2190.99	24.70	1847.60	5.16	1818.92	3.52	1755.20	-0.10	1749.92	-0.40	1748.39	-0.49
A_{14}	1707	2181.51	27.80	1834.62	7.48	1811.47	6.12	1735.37	1.66	1731.44	1.43	1730.54	1.38
A_{15}	1644	2059.16	25.25	1731.70	5.33	1720.13	4.63	1648.87	0.30	1645.10	0.07	1644.34	0.02
A_{16}	1593	1962.12	23.17	1672.62	5.00	1668.32	4.73	1617.24	1.52	1612.44	1.22	1612.27	1.21
A_{17}	1521	1846.89	21.43	1587.66	4.38	1579.95	3.88	1521.00	1.59	1541.05	1.32	1540.24	1.27
A_{18}	1501	1829.92	21.91	1555.93	3.66	1544.93	2.93	1501.00	1.18	1515.69	0.98	1517.21	1.08

Table 3: Computed values of the gas phase basicity (kJ/mol) and errors (in percentage) with respect to the experimental result obtained with the MPW1B95 density functional and different basis sets.

MPW1B95													
System	Ref. Value	B0		B1		B2		B3		B4		B5	
		Computed	Error (%)	Computed	Error (%)	Computed	Error (%)	Computed	Error (%)	Computed	Error (%)	Computed	Error (%)
A_1	1709.8	2258.25	32.08	1872.53	9.52	1839.22	7.57	1722.60	0.75	1719.98	0.60	1717.98	0.48
A_2	1723	2210.19	28.28	1846.25	7.15	1820.40	5.65	1733.03	0.58	1729.32	0.37	1725.84	0.16
A_3	1676	2114.56	26.17	1795.38	7.12	1771.47	5.70	1706.82	1.84	1702.20	1.56	1699.80	1.42
A_4	1605.8	2004.96	24.86	1684.07	4.87	1671.79	4.11	1597.14	-0.54	1595.05	-0.67	1593.87	-0.74
A_5	1722	2191.53	27.27	1827.04	6.10	1801.79	4.63	1713.64	-0.49	1710.15	-0.69	1705.68	-0.95
A_6	1722	2168.55	25.93	1817.54	5.55	1793.84	4.17	1721.30	-0.04	1717.43	-0.27	1711.62	-0.60
A_7	1697	2131.72	25.62	1697.00	5.40	1764.80	4.00	1694.49	-0.15	1691.54	-0.32	1684.65	-0.73
A_8	1692	2185.77	29.18	1817.83	7.44	1791.65	5.89	1708.65	0.98	1704.75	0.75	1701.13	0.54
A_9	1709	2162.10	26.51	1806.40	5.70	1781.96	4.27	1711.42	0.14	1707.15	-0.11	1700.94	-0.47
A_{10}	1709	2196.46	28.52	1824.10	6.73	1798.80	5.25	1715.32	0.37	1711.46	0.14	1707.71	-0.08
A_{11}	1687	2140.98	26.91	1802.87	6.87	1783.21	5.70	1700.30	0.79	1696.89	0.59	1694.33	0.43
A_{12}	1680	2091.53	24.50	1773.56	5.57	1751.10	4.23	1688.82	0.52	1685.06	0.30	1682.34	0.14
A_{13}	1719	2147.00	24.90	1801.38	4.79	1773.66	3.18	1708.30	-0.62	1703.79	-0.88	1699.78	-1.12
A_{14}	1690	2132.73	26.20	1787.84	5.79	1767.32	4.57	1690.66	0.04	1687.40	-0.15	1685.72	-0.25
A_{15}	1613	2009.07	24.55	1687.77	4.64	1675.04	3.85	1602.25	-0.67	1599.55	-0.83	1597.28	-0.97
A_{16}	1564	1911.64	22.23	1632.62	4.39	1626.29	3.98	1573.35	0.60	1569.59	0.36	1569.06	0.32
A_{17}	1499	1786.22	19.16	1535.61	2.44	1526.71	1.85	1522.62	1.58	1488.83	-0.68	1486.71	-0.82
A_{18}	1476	1731.96	17.34	1497.91	1.48	1486.52	0.71	1458.20	-1.21	1455.81	-1.37	1447.32	-1.94

Table 4: Computed values of the gas phase basicity (kJ/mol) and errors (in percentage) with respect to the experimental result obtained with the ωB97X-D density functional and different basis sets.

ωB97X-D													
System	Ref. Value	B0		B1		B2		B3		B4		B5	
		Computed	Error (%)	Computed	Error (%)	Computed	Error (%)	Computed	Error (%)	Computed	Error (%)	Computed	Error (%)
A_1	1709.8	2265.58	32.51	1875.69	9.70	1841.82	7.72	1759.30	2.90	1723.86	0.82	1722.62	0.75
A_2	1723	2218.93	28.78	1854.02	7.60	1826.95	6.03	1772.86	2.89	1736.27	0.77	1734.10	0.64
A_3	1676	2121.70	26.59	1804.06	7.64	1780.04	6.21	1748.62	4.33	1710.83	2.08	1709.72	2.01
A_4	1605.8	2019.78	25.78	1693.29	5.45	1681.11	4.69	1641.93	2.25	1606.18	0.02	1605.92	0.01
A_5	1722	2201.03	27.82	1837.02	6.68	1810.23	5.12	1757.78	2.08	1721.50	-0.03	1719.75	-0.13
A_6	1722	2179.25	26.55	1829.58	6.25	1804.54	4.79	1766.34	2.58	1729.51	0.44	1726.41	0.26
A_7	1697	2144.58	26.37	1804.21	6.32	1779.13	4.84	1744.66	2.81	1708.47	0.68	1704.34	0.43
A_8	1692	2195.76	29.77	1829.36	8.12	1801.29	6.46	1752.69	3.59	1716.03	1.42	1714.49	1.33
A_9	1709	2173.31	27.17	1820.28	6.51	1794.17	4.98	1758.18	2.88	1721.01	0.70	1717.80	0.52
A_{10}	1709	2206.11	29.09	1835.02	7.37	1807.73	5.78	1757.57	2.84	1720.81	0.69	1718.78	0.57
A_{11}	1687	2151.85	27.55	1812.06	7.41	1791.28	6.18	1743.51	3.35	1707.08	1.19	1706.38	1.15
A_{12}	1680	2100.31	25.02	1786.89	6.36	1763.65	4.98	1736.03	3.33	1699.03	1.13	1698.20	1.08
A_{13}	1719	2161.24	25.73	1816.03	5.64	1787.43	3.98	1756.18	2.16	1718.43	-0.03	1716.92	-0.12
A_{14}	1690	2151.26	27.29	1803.91	6.74	1780.65	5.36	1736.23	2.74	1699.83	0.58	1698.96	0.53
A_{15}	1613	2023.77	25.47	1697.93	5.27	1682.03	4.28	1647.69	2.15	1611.45	-0.10	1610.32	-0.17
A_{16}	1564	1940.06	24.04	1646.99	5.31	1640.74	4.91	1624.03	3.84	1584.07	1.28	1585.51	1.38
A_{17}	1499	1820.72	21.46	1563.30	4.29	1553.54	3.64	1499.00	3.51	1514.90	1.06	1513.83	0.99
A_{18}	1476	1804.55	22.26	1528.71	3.57	1519.53	2.95	1476.00	3.30	1489.02	0.88	1491.16	1.03
